# Mapping of Morphine-Induced *OPRM1* Gene Expression Pattern in the Adult Zebrafish Brain

**DOI:** 10.3389/fnana.2020.00005

**Published:** 2020-02-20

**Authors:** Mageswary Sivalingam, Satoshi Ogawa, Ishwar S. Parhar

**Affiliations:** Brain Research Institute, Jeffrey Cheah School of Medicine and Health Sciences, Monash University Malaysia, Bandar Sunway, Malaysia

**Keywords:** opiates, mu opioid receptor, *cfos*, *NPAS4a*, teleosts

## Abstract

Morphine is a potent analgesic opiate commonly used in treating pain, and it is also a substance of abuse and highly addictive. Hence, it is vital to discover the action sites of morphine in the brain to increase its efficacy of treatment. In the present study, we aimed at identifying comprehensive neuroanatomical locations that are sensitive to morphine in the adult zebrafish (*Danio rerio*). We performed *in situ* hybridization to localize the mu opioid receptor (*oprm1)* gene and to map the morphine sensitive brain areas using neuronal PAS domain-containing protein 4a (*npas4*a), an early gene marker. Real-time PCR was used to detect changes in mRNA levels of *oprm1* and *npas4*a in control and acute morphine treated fish (2 mg/L; 20 min). Intense positive *oprm1* signals were seen in the telencephalon, preoptic area, habenula, hypothalamic area and periventricular gray zone of the optic tectum. Acute morphine exposure significantly increased *oprm1* and *npas4a* mRNA levels in the medial zone of dorsal telencephalon (Dm), ventral region of the ventral telencephalon (Vv), preoptic area, and in the hypothalamus but a decrease in *oprm1* and *npas4a* signals in the dorsal habenula. This study provides a detailed map of *oprm1* localization in the brain, which includes previously unreported *oprm1* in the habenula of teleost. Presence of oprm1 in multiple brain sites implies multiple action targets of morphine and potential brain functions which could include reward, cognitive and negative emotions.

## Introduction

Morphine is a well-documented analgesic drug used to treat severe pain. In addition to its analgesic effect, morphine also exerts its rewarding properties, which leads to opioid addiction. The multiple action sites of morphine in the brain decrease the effectiveness of morphine due to development of tolerance, physical dependence, and addiction.

Morphine can bind to μ (mu) opioid receptor (MOR), δ (delta) opioid receptor (DOR) and κ (kappa) opioid receptor (KOR). However, it primarily binds to MOR to exert its analgesic pharmacological properties (Corbett et al., 1993; Kieffer, 1999). In the brain of mammals, MOR are widely expressed in several areas including the periaqueductal gray, thalamus, cingulate cortex and the insula, which is involved in mediating pain signals (Kitchen et al., 1997; Le Merrer et al., 2009; Lutz and Kieffer, 2013; Cahill et al., 2014), while MOR in the ventral tegmental area (VTA) and the nucleus accumbens (NAc) are involved in opiate-induced reward (Contet et al., 2004). The reinforcement effect of morphine is triggered by activating dopamine release from the VTA and the NAc (Hyman and Malenka, 2001; Ballantyne and LaForge, 2007). Expression of MOR is also reported in the habenula—interpeduncular nucleus (IPN) pathway; suggesting the potential role of MOR in mediating the positive and negative effect of opioids, which needs to be further investigated (Gardon et al., 2014). The current therapeutic medications manage opioid relapse, yet overdose fatalities and recurrent cases of substance abuse are increasing (King et al., 2014; McLaughlin et al., 2017). Hence, identifying the comprehensive distribution of MOR is important to elucidate the target sites and understanding of brain regions that are potentially affected by morphine.

Various regulatory molecules and intracellular signaling pathway have been implicated in the regulation of MOR expression; therefore, along with the receptor expression profile, the neural activation can provide complementary details on the brain region that are sensitive to morphine. Several electrophysiological studies in mammalian species have demonstrated the neural activation of specific brain regions or cell types in response to morphine (Iwatsubo and Clouet, 1977; Nowycky et al., 1978; Matthews and German, 1984; Diana et al., 1999; Su et al., 2012; Bull et al., 2017). Mapping of neuronal activation sites in response to morphine has also been examined using neuronal activation markers, such as c-Fos, which is an immediate early gene and extracellular signal-regulated kinase (Chang et al., 1988; Hayward et al., 1990; Valjent et al., 2004). Although most neuronal activation markers are induced by a variety of stimuli, their expression is mainly induced as a result of molecular signaling for normal cellular function and survival and not solely due to the neural activation (Ramanan et al., 2005; Ramamoorthi et al., 2011). Recently, neuronal PAS domain protein 4 (Npas4) has been identified as a brain-enriched transcription factor, which is selectively coupled to neuronal activity and can provide accurate neural activation profile (Lin et al., 2008). Although MOR distributions have been demonstrated in several mammalian and in non-mammalian vertebrates, their expression pattern and neural activation sites upon opioid treatment are less studied.

The zebrafish (*Danio rerio*) possesses the MOR-homologous gene (*oprm1*, also known as ZFOR2) (Barrallo et al., 2000) with similar pharmacological characteristics to MOR in mammals (de Velasco et al., 2009). Although the distribution of the *oprm1* gene and protein in the whole tissue has been demonstrated in larval zebrafish (Bretaud et al., 2007; Sanchez-Simon and Rodriguez, 2008; Arévalo et al., 2018), but their detailed expression patterns in the adult brain remains unreported. In the present study, we first examined the expression sites of the *oprm1* gene in the brain of adult zebrafish using *in situ* hybridization. Next, to identify brain regions sensitive to morphine, we examined the effect of acute (20-min) morphine exposure on *oprm1, cfos* and *npas4a* gene expression in the brain by *in situ* hybridization and real-time PCR.

## Materials and Methods

### Animal and Housing

Sexually mature male (4–6 months, 0.5–1.0 g body weight), the RIKENWako (RW) wild-type zebrafish (*Danio rerio*; RRID: ZDB-GENO-030619-2), obtained from the National BioResource Center of Japan (www.shigen.nigac.jp/zebra/) were maintained in freshwater aquaria at 27 ± 0.5°C under a controlled natural photo-regimen (14/10 h, light/dark phase). The fish were fed with an Adult Zebrafish Diet (Zeigler, Gardners, PA, USA) twice daily. The fish were maintained, and all the experiments were carried out under the guideline of the Animal Ethics Committee of Monash University (ethics approval number: MARP/2017/049). However, this study is not pre-registered.

### Acute Morphine Treatment

To reduce handling stress, fish were acclimatized for a week before the treatment. Fish were then assigned randomly to acute morphine treatment or control; no particular randomization method was performed to allocate the subjects in groups in this study. Fish were individually treated by immersion in a tank [sized 361 mm (L) × 218 mm (W) × 256 mm (D)] with water containing 2 mg/L morphine sulfate pentahydrate (Lipomed AG, Switzerland) for 20 min. The morphine treatment procedure was chosen based on a protocol previously reported (Stewart et al., 2011). As morphine is an analgesic, it does not cause any form of pain or suffering to the fish, and the doses used are those utilized in previous studies that do not cause toxicity (Magalhães et al., 2017; Chatigny et al., 2018). Therefore, no major effects from the treatments on fish were expected and only normal healthy fish were used in this study. After the treatment, the fish were anesthetized by immersion in water containing benzocaine (0.1 g benzocaine/200 mL, Sigma) and the brain was dissected for *in situ* hybridization and real-time PCR analysis. The same treatment protocol was employed for control samples, but they were immersed 20 min in water without morphine. In both the morphine and control group, the treatments were carried out simultaneously on separate immersion tanks from 1,400 to 1,600 h.

### *In situ* Hybridisation of Zebrafish *oprm1, cfos*, and *npas4a* Genes

The sense and antisense digoxigenin (DIG) labeled-riboprobes for *oprm1, cfos* and *npas4a* were transcribed from a pGEM T-Easy vector (Promega, Madison, WI) containing 1,180, 438, and 737 bp fragments of zebrafish cDNA [GenBank accession numbers: NM_131707, NM_205569.1, and NM_001045321, respectively; the National Center for Biotechnology Information (NCBI, RRID: nif-0000-00139)]. DIG labeling was achieved using MAXIscript (Cat# AM1322M, Ambion, Austin, TX) and DIG RNA labeling mix (Cat# 11277073910, Roche Diagnostics, Mannheim, Germany) following the manufacturers' instructions. The brain samples were fixed in buffered 4% paraformaldehyde for 6 h at 4°C, cryoprotected in 20% sucrose solution, and embedded in Tissue Tek OCT compound (Sakura Finetechnical, Tokyo, Japan). The specificity of the probes were examined using sagittal sections (*n* = 2 for each gene). Coronal sections (*n* = 6 per group for each gene) were used to examine the detailed expression of the genes. Brain sections (14 μm thickness) were cut in a cryostat and thaw-mounted onto 3-aminopropylsilane (APS)-coated glass slides. DIG-*in situ* hybridization was performed as described previously (Ogawa et al., 2012). Briefly, the sections were permeabilised with 0.2 M HCl and then treated with proteinase K (1 μg/mL) for 15 min, and hybridized with DIG-labeled riboprobes (50 ng/mL) at 55°C overnight in a humidified chamber. Following hybridization, the sections were washed and blocked with 2% normal sheep serum. The DIG-labeled probes then detected with an alkaline phosphatase-conjugated anti-DIG antibody (Roche Cat# 11093274910, RRID: AB_514497, diluted 1:500). For the localization of *oprm1, cfos*, and *npas4a* probe expressions, the chromogenic reaction was achieved with 4-nitroblue tetrazolium chloride/5-bromo-4-chloro-3-indolyl-phosphate (NBT/BCIP, Roche Cat# 11681451001). To examine the effect of morphine on *oprm1*, the signals were amplified using Tyramide Signal Amplification (TSA) Plus Fluorescein kit (Perkin-Elmer, Wellesley, MA) according to the manufacturer's instructions. Preliminary evaluation showed no difference in localization and staining intensity of Dig-labeled *cfos* expressing cells. Therefore, in subsequent experiments npas4a and not c-fos was used as a neuronal activity marker.

### Image Capturing, Cell Counting, and Statistical Analysis

The DIG-stained sections were cover-slipped, scanned, and the images were then captured with a Zeiss MIRAX Midi Slide scanning system (Cat# 000000-1496-488, Zeiss, Göttingen, Germany) at a resolution of 230 nm using a ×20 objective and processed with the Mirax Viewer Image Software (3DTech, Budapest, Hungary). To standardize sections with different background intensity, all the section were changed to gray mode using adobe illustrator software CS5.1. For the manual counting of the number of DIG-labeled *npas4a* expressing cells (control *n* = 6, acute morphine-treated *n* = 6), an average of 10 consecutive sections/region for each sample were used. Single blinding procedure was used to count the cell number. No sample calculation was performed to predetermine the sample size. However, the sample size in this study is comparable to that in the previous study on neuronal activity quantification in zebrafish (Lau et al., 2011). Numbers of cells expressing *npas4a* were counted in regions showing prominent changes including the dorsal and ventral telencephalon (155 mm^2^), anterior preoptic area (186 mm^2^), posterior preoptic area (49 mm^2^), habenula (85 mm^2^), and the hypothalamic region (160 mm^2^). Cell counting was not performed in periventricular gray zone of optic tectum (PGZ) due to the high density and compact nature of *npas4a* cells in this area. All cell counting data were analyzed using the IBM statistical package V22.0 (IBM SPSS Statistics for Windows, Version 22.0. Armonk, NY: IBM Corp). Independent *t*-test was used for comparison between control and treated groups. Data are expressed as mean ± SEM with the significance set at *P* < 0.05. While for the semi-quantitative analysis of *oprm1* and *npas4a* mRNA, staining density was subjectively scored on a five-point scale as follows + + + (high), + + (moderate), + (low), and – (absent). Nomenclature for the zebrafish brain regions was adopted from (Wullimann et al., 1996; Rink and Wullimann, 2001; Mueller et al., 2004; Mueller and Guo, 2009; Liu et al., 2015).

### Quantification of *oprm1* and *npas4a* mRNA Levels in the Macrodissected Brain Regions Using Real-Time PCR

Gene expression levels of *oprm1* and *npas4a* were examined by real-time PCR in both the control and acute morphine-treated group (*n* = 20; control group = 10, treated = 10). Using forceps and under the stereoscopic microscope, the brain was divided into six regions comprising (i) the olfactory bulb, (ii) telencephalon including the preoptic area and habenula, (iii) hypothalamic region, (iv) optic tectum, (v) cerebellum, and (vi) hind brain region as shown in **Figure 3A**. The total RNA was isolated from six brain regions using 100 μl of Trizol (Life Technologies, Gaithersburg, MD) reagent according to the manufacturer's instruction. The extracted total RNA was dissolved in 20 μl diethylpyrocarbonate-treated water (DEPC-MQ), and RNA concentration and purity were quantified using NanoDrop Spectrophotometer ND 1000 (Thermo Fisher Scientific). One microgram of total RNA was then reverse transcribed to cDNA using the High Capacity cDNA Reverse Transcription kit (Applied Biosystems) in a 20-μl reaction mixture containing 1× RT buffer, 1× deoxynucleotide triphosphate mix, 1× RT random primers, 20 U of ribonuclease inhibitor, and 10 U of MultiScribe Reverse Transcriptase. The cDNA was then subjected to real-time PCR for *oprm1, npas4a*, and β*-actin* (*actb1*, reference gene) (GenBank accession no NM_131707, NM_001045321, and NM_131031). The primer sequences were as follows: *oprm1*; F: 5′-ATGGGACTGGTGGGAAACG-3′ and R: 5′-GCCAAGGAATCTGCTAGAGCAA-3′, *npas4a*; F: 5′-CCTGGGGCAACAACCTGA-3′ and R:5′-CTTCCACTCCCATCTTTGCGG-3′, and β*-actin*; F: 5′-AGAGCTATGAGCTGCCTGACG-3′ and R: 5′-CCGCAAGATTCCATACCCA-3′. The total 10 μl PCR reaction mixture contained 2× SensiFAST SYBR Hi-ROX Kit Mix (Bioline), 0.1 μM each of forward and reverse primers (Table 1), and 1 μl of sample cDNA, which was analyzed using an ABI PRISM 7500 Sequence Detection System (Applied Biosystems). DEPC-MQ was used as a negative control (non-template control). The reaction program consisted of 50°C for 2 min, 95°C for 10 min, and 40 cycles of 95°C for 15 s and 60°C for 1 min, followed by a dissociation stage. The threshold cycle (Ct) of each gene was determined and normalized to the β*-actin* mRNA levels. The data were then analyzed according to the relative gene expression calculated by 2^−ΔΔCt^. All data are presented as means ± SEM, and statistical analyses were performed using an independent *t*-test to observe comparisons between control and morphine-treated fish.

**Table 1:** Expression of *oprm1* mRNA in the brain of adult zebrafish.

**Brain regions**	**ISH staining distribution**			**Brain regions**	**ISH staining distribution**		
	***OPRM1***				***OPRM1***		
	**Abbr**	**Control**	**Treated**		**Abbr**	**Control**	**Treated**
**Telencephalon**							
**Olfactory Bulbs**	OB			**Synencephalon**			
Lateral olfactory tract	LOT	+	+	Nuclues of MLF	NMLF	++	++
Medial olfactory tract	MOT	+	+	Periventricular gray zone of optic tectum	PGZ	+++	+++
Primary olfactory fiber layer	POF	–	–	Periventricular pretectal nucleus, dorsal part	PPd	+	+
Glomerular layer of olfactory bulb	GL	+	+	Periventricular pretectal nucleus, ventral part	PPv	++	+++
External cellular layer of olfactory bulb	ECL	++	++				
Internal cellular layer of olfactory bulb	ICL	++	++	**Mesencephalon**			
**Dorsal telecephalic area**				**Tectum Opticum**			
Dorsal telecephalic area	D	++	++	Optic tectum	TeO	++	+++
Dorsal zone of D	Dd	+	+	Torus longitudinalis	TL	+++	+++
Lateral zone of D	Dl	++	++	Commisura tecti	Ctec	–	–
Central zone of D	Dc	+	+	**Torus semicircularis**			
Medial zone of D	Dm	++	+++	Central nucleus of torus semicircularis	TSc	+	+
Posterior zone of D	Dp	+	+	Ventrolateral nucleus of torus semicircularis	TSvl	+	+
Nucleus taeniae	NT	+	+	**Tegmentum**			
				Dorsal tegmental nucleus	DTN	+	++
**Ventral telecephalic area**	V			Nucleus of the lateral lemniscus	NLL	+	++
Central nucleus of V	Vc	–	–	Nucleus Interpeduncularis	Nin	++	++
Dorsal nucleus of V	Vd	++	++	Vascular lacuna of area postrema	Vas	+	+
Lateral nucleus of V	Vl	–	–	Superior reticulam formation	SRF	+	+
Ventral nucleus of V	Vv	+++	+++				
Supracommissural nucleus of V	Vs	++	++	**Rhombencephalon**			
Postcommissural nucleus of V	Vp	++	++	Cerebellum			
Entopeduncular nucleus, dorsal part	End	+	+	Eminentia granularis	EG	++	++
Entopeduncular nucleus, ventral part	ENv	+	+	Corpus cerebelli	Cce	++	++
				Vavula cerebelli			
				medial	Vam	+	+
**Diencephalon**				lateral	Val	+	+
**Preoptic area**				Commmsura cerebelli	Ccer	+	+
Diencephalic ventricle	DiV	–	–	**Medulla oblongata**			
parvocellular preoptic nucleus, anterior part	PPa	+++	+++	Medial longitudinal fascicle	MLF	+	+
parvocellular preoptic nucleus, posterior part	PPp	+++	+++	Ventrolateral longitudinal fascicle	Nvmv	+	+
Suprachiasmatic nucleus	SC	+	+	Superior raphe nucleus	SR	++	++
Magnocellular preoptic nucleus	PM	+	+	Griseum centrale	GC	+	+
**Epithalamus**							
Dorsal Habenula	dHb	++	+				
Ventral Habenula	vHb	+	+				
**Dorsal Thalamus**							
Central posterior thalamic nucleus	CP	+	+				
Anterior thalamic nucleus	A	+	+				
**Ventral Thalamus**							
Intermediate thalamic nucleus	I	+	+				
Ventrolateral thalamic nucleus	VL	+	+				
Ventromedial thalamic nucleus	VM	+	+				
**Posterior tuberculum**							
Posterior tuberal nucleus	PTN	+	++				
Lateral preglomerular nucleus	PGl	+	++				
Medial preglomerular nucleus	PGm	+	++				
Torus lateralis	Tla	+	++				
**Hypothalamus**							
Dorsal zone of periventricular hypothalamus	Hd	+	++				
Ventral zone of periventricular hypothalamus	Hv	+	++				
Anterior tuberal nucleus	ATN	+	++				
Lateral hypothalamic nucleus	LH	+	++				
Paraventricular organ	PVO	–	–				

*The chart is showing the summary of oprm1 mRNA expressing brain regions based on the data collected from alternate coronal sections labeled with oprm1 mRNA expressing cells*.

*Symbols indicate relative expression of oprm1 mRNA: + + + (high), ++ (moderate), + (low), and – (absent). Abbreviation represented by Abbr*.

## Results

### DIG-*in situ* Localization of *oprm1* mRNA in the Brain

**Figure 1:**
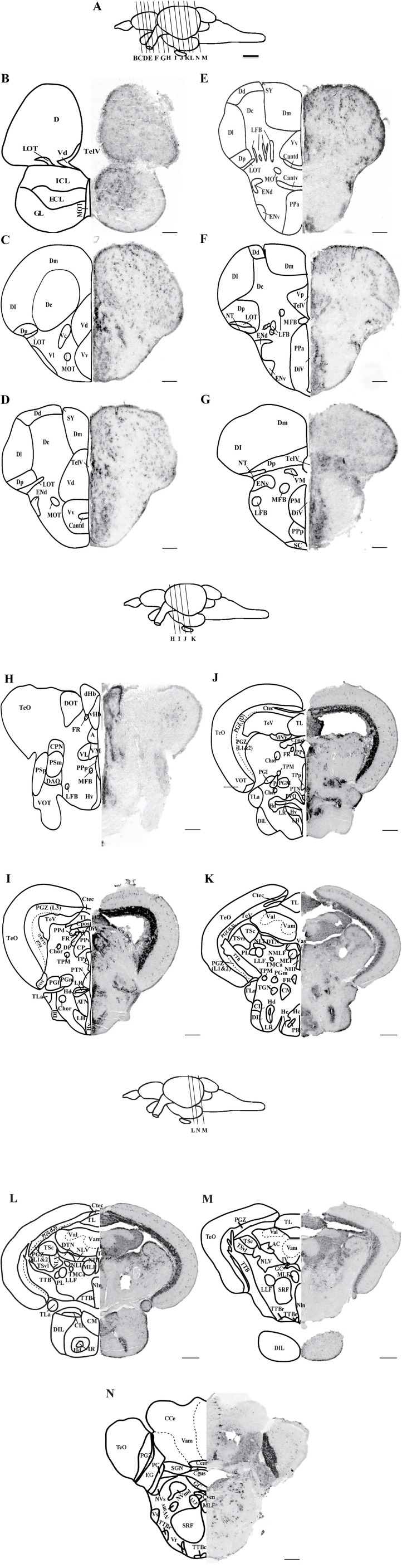
Localization of *oprm1* mRNA expression in the brain of zebrafish. Schematic coronal drawing of the brain of zebrafish showing the distribution of *oprm1* mRNA expression. **(A)** Lines on a schematic sagittal drawing view of the zebrafish brain indicate levels of coronal sections, **(B–N)** coronal sections showing the distribution of *oprm1* mRNA. *n* = 3, Scale bars: **B–G** (100 μm) and **H–N** (200 μm).

**Figure 2:**
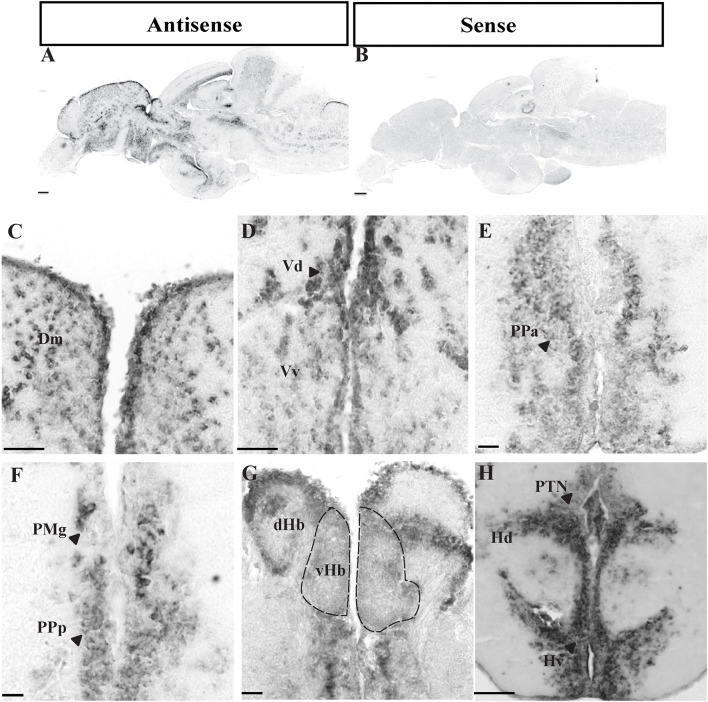
Expression of *oprm1* mRNA in the brain of zebrafish. Photomicrographs showing expression of *oprm1* genes in sagittal **(A,B)** and coronal sections **(C–H)** of zebrafish brain. **(A)** Antisense probe for oprm1 gene showed positive signal and **(B)** Sense probes for *oprm1* gene showed no signals. **(C)** Medial zone of dorsal telencephalic area, **(D)** Dorsal and Ventral nucleus of ventral telencephalic area, **(E,F)** Anterior and posterior part of parvocellular preoptic area, **(G)** Habenula area, and **(H)** Periventricular hypothalamic area. *N* = 3, Scale bars: **A–B** (100 μm) and **C–H** (50 μm).

DIG-*in situ* hybridization showed expression of *oprm1* throughout the zebrafish brain, which includes the telencephalon, diencephalon, mesencephalon, and the rhombencephalon, as summarized in Table 1 and illustrated in Figure 1. Sense probes did not show any signals (Figure 2) confirming the specificity of the antisense probes.

#### Telencephalon

Although strong positively labeled *oprm1* cells was observed in the internal and external cellular layer of olfactory bulb, relatively diffused distribution of *oprm1* signals were also expressed (Figure 1B, Table 1). In the ventral telencephalon, *oprm1* expressing cells lie in the dorsal and ventral nucleus of the ventral telencephalon (Figures 1C,D, 2D, Table 1). However, staining intensity in the ventral nucleus was weaker compared to the dorsal region of ventral telencephalon. Cells with strong signals were observed in almost all dorsal telencephalon subdivisions including the dorsal, lateral, medial, central, and posterior zone of dorsal telencephalic area (Figures 1B–G, Table 1). Strong positive signals were also observed in the dorsal and ventral part of the entopeduncular nucleus (Figures 1E–G, Table 1).

#### Diencephalon

Cells expressing *oprm1* with intense staining were seen in the anterior, posterior parvocellular preoptic area and the magnocellular preoptic nucleus (Figures 1F,G, 2E,F, Table 1). In the epithalamus, cells expressing *oprm1* lie in the dorsal and ventral habenula; however, the staining was quite diffuse (Figures 1H, 2G, Table 1). In the hypothalamic region, cells expressing *oprm1* lie in the dorsal and ventral zone of the periventricular hypothalamus, lateral hypothalamic nucleus, and the posterior tuberal nucleus (Figures 1I, J, 2H, Table 1).

#### Mesencephalon

Cells expressing *oprm1* lie in the optic tectum and the torus longitudinalis (Figure 1J). Strong signals were observed in the PGZ, however, within the PGZ nucleus and between sections containing the PGZ nucleus there was variations in signal intensity. In the tegmentum area, *oprm1* expression was observed in the oculomotor nucleus and dorsal tegmental nucleus (Figure 1K).

#### Rhombencephalon

In the cerebellum, cells expressing *oprm1* lie in several regions, such as the corpus cerebella, Eminentia granularis and few cells in lateral and medial division of valvula cerebelli (Figure 1N, Table 1). Some cells expressing *oprm1* were also found in the medulla oblongata, such as the medial longitudinal fascicle and central gray (Figures 1M,N, Table 1).

### Effect of Morphine on *oprm1* Expression in the Brain of Zebrafish

**Figure 3:**
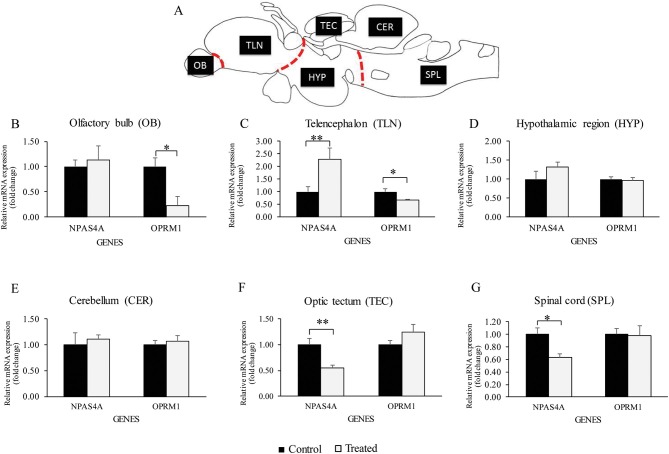
Relative *oprm1* and *npas4a* mRNA expression in the brain. **(A–G)** Graphs showing the relative gene expression (fold change) of *oprm1* and *npas4a* mRNA expression after exposure to acute vehicle (Control) and morphine via water immersion (Treated) across several brain region. **(A)** Schematic sagittal drawing of macro-dissected brain region, **(B)** olfactory bulb region, **(C)** Telencephalon region, **(D)** Hypothalamic region, **(E)** Cerebellum, **(F)** Optic tectum, **(G)** Spinal cord region. Data are presented as mean ± SEM. Independent *t*-test comparisons between control and morphine-treated fish. *P < 0.05; **P < 0.01 vs. controls (*n* = 6).

Using Real-time PCR significant down-regulation of *oprm1* mRNA was seen in the olfactory bulb and telencephalon in morphine treated fish (Figures 3B,C). However, morphine had no effect on *oprm1* mRNA in other brain regions (Figures 3D–G).

**Figure 4:**
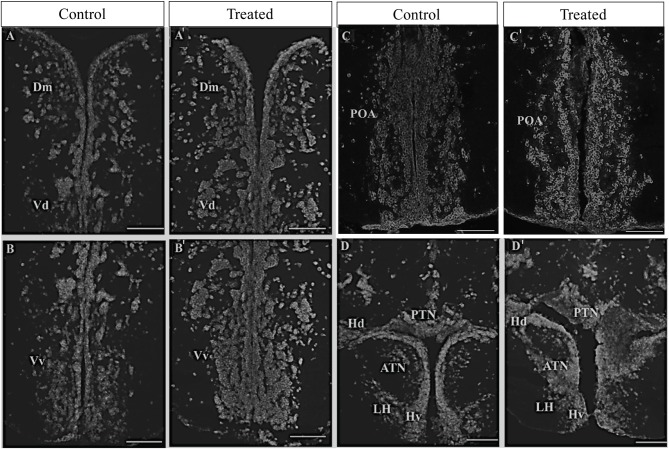
Effect of morphine on *oprm1* mRNA expression in the brain of zebrafish. Fish were individually immersed in morphine 2 mg/L (treated) or water (control) for 20 min and then the brain samples were collected. **(A–D′)** Photomicrographs showing expression of *oprm1* genes. (**A**; control and **A′**; morphine treated) Medial zone of the dorsal telencephalic area and dorsal nucleus of the ventral telencephalic area, **(B,B′)** ventral nucleus of the ventral telencephalic area, **(C,C′)** posterior part of preoptic area, and **(D,D′)** ventral periventricular hypothalamus. *N* = 6 each group. Scale bars: **(A–D)** and **(A′-D′)** (100 μm).

The effect of morphine on the *oprm1* gene expression was further morphologically assessed via *in situ* hybridization (ISH). In morphine-exposed fish, ISH showed high signal intensity of *oprm1* in the medial zone of dorsal telencephalon (Dm) and in the dorsal and ventral region of the ventral telencephalon (Vv and Vd) as compared to controls [Figure 4A (control), Figure 4A′ (morphine treated), Figures 4B,B′]. Similarly, in the posterior part of the parvocellular preoptic nucleus and periventricular hypothalamic region, higher signal intensity of *oprm1* was observed in morphine-treated fish as compared to control fish (Figures 4C,C′,D,D′).

### Effect of Morphine on *Npas4a* Expression in the Brain of Zebrafish

Real-time PCR showed a significant increase in npas4a mRNA levels in the telencephalon (Figure 3C), but a decrease in the optic tectum and spinal cord in morphine treated fish (Figures 3F,G). There was no effect of morphine treatment on *npas4a* mRNA levels in other brain regions (Figures 3B,D,E).

**Figure 5:**
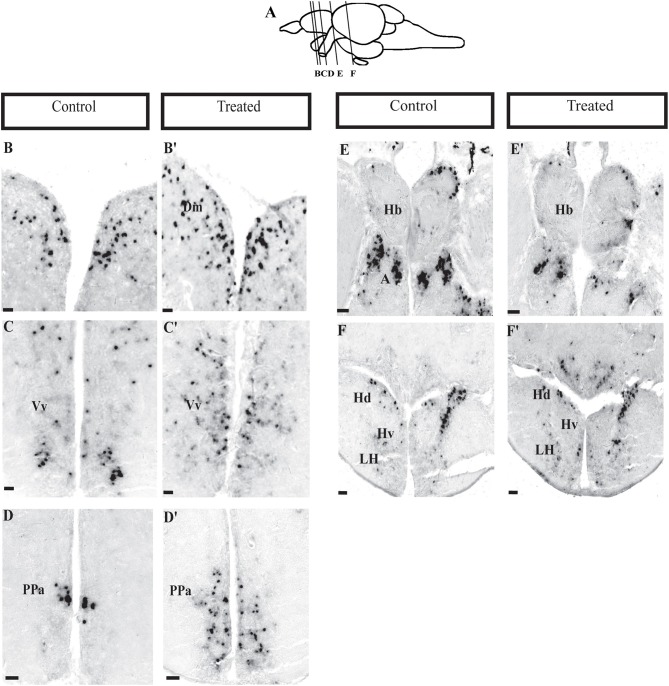
Expression of *npas4a* gene in the brain of zebrafish. Photomicrographs showing the mRNA expression of neural activity marker, *npas4*. **(A)** Lines on the schematic sagittal drawing view of the zebrafish brain indicate levels of coronal sections (**B**; control and **B′**; morphine treated) medial zone of dorsal telencephalic area, **(C,C′)** ventral nucleus of ventral telencephalic area, **(D,D′)** preoptic area, **(E,E′)** habenula, and **(F,F′)** ventral periventricular hypothalamus. *N* = 6 each group. Scale bars: **(B–F)** and **(B′-F′)** (100 μm).

**Figure 6:**
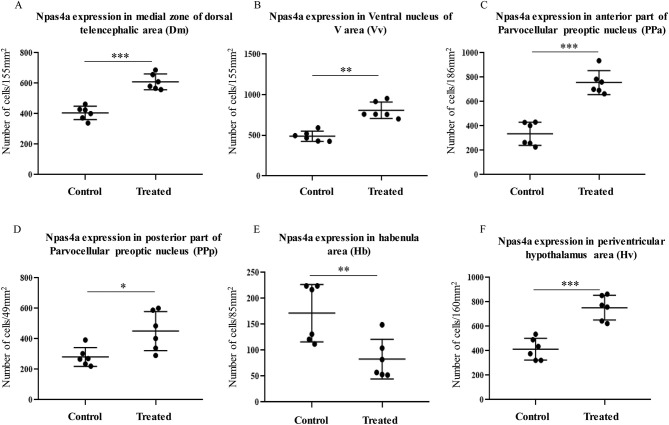
Number of *npas4* mRNA expressing cells in control and morphine-treated fish. **(A–F)** Graphs showing the difference in *npas4a* mRNA expression via cell counting after exposure to acute vehicle (control) and morphine via water immersion. **(A)** Medial zone of the dorsal telencephalic area, **(B)** ventral nucleus of the ventral telencephalic area, **(C,D)** anterior and posterior part of parvocellular preoptic area, **(E)** habenula, and **(F)** ventral periventricular hypothalamus. Data are presented as mean ± SEM. Independent *t*-test comparisons between control and morphine-treated fish. *P < 0.05; **P < 0.01, ***P < 0.001 vs. controls (*n* = 6).

*In situ* hybridization, showed a significant changes in *npas4a* expression in several brain areas (Figure 5A). Increase in *npas4a* signal intensity were observed in the medial zone of dorsal and ventral nucleus of telencephalon, preoptic area and hypothalamus [Figures 5B–D,F (control), Figures 5B′-D′,F′ (morphine treated)], but a decrease in the dorsal habenula and in the anterior thalamic nucleus in morphine treated fish (Figure 5E). *npas4a* expressing cell numbers were significantly increase in the medial region of the dorsal telencephalon (Figure 6A), ventral region of the ventral telencephalon (Figure 6B), anterior and posterior part of the parvocellular preoptic nucleus (Figures 6C,D), and the periventricular hypothalamus area (Figure 6F) but cell numbers were significantly decreased in the dorsal habenula (Figure 6E).

## Discussion

In the present study, we investigated the distribution of cells expressing *oprm1* mRNA in the brain of adult zebrafish. The ISH staining demonstrated wider distribution of *oprm1* mRNA expression throughout the brain, which is similar to *oprm1* expression that were previously demonstrated in the larval zebrafish brain using whole-mount *in situ* hybridization and a MOR antibody generated specific to zebrafish (Sanchez-Simon and Rodriguez, 2008; Arévalo et al., 2018). Although the distribution of MOR has been reported in the brain of several teleost species (Chadzinska et al., 2009; Stevens, 2009), this report is the first detailed mapping of MOR in the adult brain of a teleost, the zebrafish.

Previous studies in zebrafish larvae using whole-mount ISH have shown positive MOR signals and also immunoreactive cells in the telencephalon region (Sanchez-Simon and Rodriguez, 2008; Arévalo et al., 2018), likewise, in this study, we observed intense *oprm1* positive signals in the dorsal and ventral telencephalon. In fish (rainbow trout, lamprey, eel, and coho salmon) (Bird et al., 1988; Ebbesson et al., 1996), and in birds (Reiner et al., 1989) MOR binding sites have been reported in the telencephalon. Similarly, in mammals MOR signals have been observed in telencephalic nuclei, which include nucleus accumbens, striatum, amygdala, and the hippocampus (Delfs et al., 1994; Mansour et al., 1994). These studies suggest the distribution of MOR in the telencephalon is well-conserved across vertebrate species, which could be a center for reward (Charbogne et al., 2017; Ben Hamida et al., 2019).

Positive *oprm1* signal have been observed in the diencephalon of larvae zebrafish (Sanchez-Simon and Rodriguez, 2008), anterior preoptic area of rainbow trout (Bird et al., 1988) and medial preoptic area of rodents (Kaufman et al., 1995; Gulledge et al., 2000). Similarly, the present study shows a wide distribution of intense *oprm1* signals in the anterior and posterior preoptic area. Interestingly, we observed oprm1 expression in the dorsal habenula similar to that in the medial habenula of rodents (Mansour et al., 1987, 1994; Gardon et al., 2014). There are no reports of MOR binding sites or MOR expression in the habenula of other non-mammalian vertebrates, including fish (Bird et al., 1988). The lack of MOR in the habenula of non-mammalian vertebrates could be due to low expression of the receptor, which could not be detected by ISH and radio-ligand binding assay, it could be an oversight or a complete absence of the receptor. MOR in the habenula has been implicated in mediating analgesia, aversive and reward functions in rodents. Since the mammalian medial habenula is homologous to the dorsal habenula in the zebrafish, this function of MOR could be conserved.

The current study also showed intense *oprm1* positive signals in the optic tectum in particular in the periventricular gray zone of optic tectum (PGZ). In the optic tectum, immunoreactive cells have been reported in zebrafish larvae (Sanchez-Simon and Rodriguez, 2008), and MOR binding sites in the rainbow trout (Bird et al., 1988). In rodents, moderate expression of MOR has been reported in the superior colliculus, which is referred to as the optic tectum in non-mammalian vertebrates. Since the optic tectum is the site of neurogenesis in teleost (D'Angelo et al., 2014; Cacialli et al., 2016) and is involved in visual sensory functions, it is possible that MOR might be related to these functions.

In the present study, npas4a induced neuronal activity was seen within 20 min of morphine exposure. Therefore, in the zebrafish *npas4a* compared to *c-fos*, which takes 30–90 min (Lau et al., 2011; Sun and Lin, 2016) is probably a more reliable neuronal activity marker to map morphine sensitive brain regions. Interestingly, in our study, a single exposure to morphine was sufficient to activate *npas4a* expression in the telencephalic and diencephalic neuronal population. However, in rodents multiple and not single dose (subthreshold stimulus) of psychostimulant (Guo et al., 2012), except a single acute injection of cocaine are needed to activate *npas4* (Ye et al., 2016). Morphine has poor lipid solubility (Newby et al., 2009); however, for adult zebrafish a single immersion in morphine solution for 30 min increased brain morphine concentration (Lau et al., 2006). Furthermore, adult zebrafish immersed in a solution containing butorphanol, a molecule chemically similar to morphine, exhibit significant analgesic effect (Schroeder and Sneddon, 2017), while zebrafish larvae immersed in a solution of morphine exhibit noxious stimulus (Lopez-Luna et al., 2017). In addition, blocking MOR using naloxone (MOR antagonist) reduces place preference behavior to morphine in both adult and larval zebrafish (Lau et al., 2006; Bretaud et al., 2007). These studies suggest that sufficient amounts of morphine reach all brain structures and provide evidence of selective binding of morphine to activate oprm1 in the zebrafish.

Following acute morphine exposure, *oprm1* mRNA levels in macro-dissected olfactory bulb and telencephalon were significantly down-regulated, while ISH semi-qualitative signal analysis showed an increase in *oprm1* in the telencephalon. On the other hand, *npas4a* mRNA expression and *npas4a* expressing cell numbers were increased in the telencephalon. Similarly, in rats, acute morphine treatment increases MOR density and neuronal activity in the forebrain especially in the nucleus accumbens (Haberstock-Debic et al., 2003), which is homologous to the dorsal nucleus of ventral telencephalon region (Vd) in teleost (O'Connell and Hofmann, 2011). The discrepancies between *in situ* hybridization and RT-PCR results may be due to limitations of the two techniques. Results obtained with the RT-PCR are from dissected small brain regions with a risk of dissection error and hence high variability in results. On the other hand, *in situ* hybridization is a sensitive method that allows detail anatomical localization, but the analysis of signal intensity is semi-quantitative.

Morphine treatment did not affect *oprm1* and *npas4a* mRNA levels in the diencephalon but an increase in *oprm1* and *npas4a* ISH signal and *npas4a* expressing cells were seen in the preoptic and hypothalamic area. Similarly, in female rats, oprm1 mRNA expression was increased in the preoptic-hypothalamic area upon morphine treatment (Petersen and LaFlamme, 1997; Šlamberová et al., 2005). The presence and upregulation of *oprm1* gene together with *npas4a* expression in the preoptic-hypothalamic nuclei suggest the possibility of morphine interaction with the neuroendocrine and the monoamine systems in this brain region (Yu et al., 1991; Bellipanni et al., 2002; Prasad et al., 2015). In the habenula, morphine induced downregulation of *oprm1* and *npas4a* expression. Although there are no reports on the regulation of MOR in the habenula of rodents, a reduced neuronal activity was reported in the previous study (Hashimoto et al., 2009); which is associated with anti-nociceptive effect and could potentially be related to many of habenula functions, such as negative emotions.

In the hindbrain, acute morphine treatment caused no change in *oprm1* mRNA levels. However, more specific ISH staining showed an increase in *oprm1* signal in the optic tectum area, torus longitudinalis and tegmental area. Meanwhile, acute morphine treatment down-regulates *npas4a* mRNA levels in the optic tectum and spinal cord but induction of npas4a was seen in the ventral entopeduncular nucleus, interpeduncular nucleus and Corpus cerebelli in the cerebellum, where very low expression of oprm1 was seen. Expression of npas4 function to promote a reduction in overall circuit activity through the late response genes (Spiegel et al., 2014). In rodents, morphine treatment could down-regulate glutamate transporter in the hindbrain and spinal cord (Mao et al., 2002). This suggests that several neuronal groups in the hindbrain might be regulated by morphine through indirect pathways. It should be noted that the activation or inhibition of opioid receptor signaling is associated with the different G protein interactions. Previous studies showed that the activation of the G-coupled protein receptor has led to decrease of neurotransmitter release or membrane potential hyperpolarization, and indirectly mediate morphine action (Mazei-Robison and Nestler, 2012; Cachope and Pereda, 2015). Thus, further analysis for G protein signaling pathways are necessary to interpret specific activation or inhibition of *npas4a*-expressing cells.

## Summary

In the present study, we found specific cell populations that are sensitive to morphine. The wide distribution of *oprm1* gene, as well as morphine-dependent expression of *npas4a* in the brain, indicate that morphine can act on multiple brain sites and neural circuits, which implies that morphine can potentially influence a variety of brain functions including reward, cognitive, and aversive functions in fish.

## Data Availability Statement

The datasets generated for this study are available on request to the corresponding author.

## Ethics Statement

The animal study was reviewed and approved by Animal Ethics Committee of Monash University (ethics approval number: MARP/2017/049).

## Author Contributions

MS, SO, and IP designed the research. MS performed the research and wrote the paper. SO and MS analyzed the data. SO and IP edited the manuscript.

### Conflict of Interest

The authors declare that the research was conducted in the absence of any commercial or financial relationships that could be construed as a potential conflict of interest.

## Abbreviations

*OPRM1*: opioid receptor mu 1
*NPAS4a*: neuronal PAS domain protein 4a
MOR: mu opioid receptor
VTA: ventral tegmental area
NAc: nucleus accumbens.
